# Use of the Relaxometry Technique for Quantification of Paramagnetic Ions in Aqueous Solutions and a Comparison with Other Analytical Methods

**DOI:** 10.1155/2016/8256437

**Published:** 2016-05-17

**Authors:** Bruna Ferreira Gomes, Juliana Soares da Silva Burato, Carlos Manuel Silva Lobo, Luiz Alberto Colnago

**Affiliations:** ^1^Instituto de Química de São Carlos, Universidade de São Paulo, Avenida Trabalhador São-Carlense 400, 13560-070 São Carlos, SP, Brazil; ^2^Embrapa Instrumentação, Rua XV de Novembro 1452, 13560-970 São Carlos, SP, Brazil

## Abstract

We have demonstrated that the relaxometry technique is very efficient to quantify paramagnetic ions during* in situ* electrolysis measurements. Therefore, the goal of this work was to validate the relaxometry technique in the determination of the concentration of the ions contained in electrolytic solutions, Cu^2+^, Ni^2+^, Cr^3+^, and Mn^2+^, and compare it with other analytical methods. Two different NMR spectrometers were used: a commercial spectrometer with a homogeneous magnetic field and a home-built unilateral sensor with an inhomogeneous magnetic field. Without pretreatment, manganese ions do not have absorption bands in the UV-Visible region, but it is possible to quantify them using relaxometry (the limit of quantification is close to 10^−5^ mol L^−1^). Therefore, since the technique does not require chemical indicators and is a cheap and robust method, it can be used as a replacement for some conventional quantification techniques. The relaxometry technique could be applied to evaluate the corrosion of metallic surfaces.

## 1. Introduction

Recently we demonstrated that time domain nuclear magnetic resonance (TD-NMR) is a very efficient and robust technique to quantify paramagnetic ions in a solution during* in situ* measurements of electrodeposition reactions [1, 2]. It is also efficient in determining the solubility constant, *K*_sp_, of paramagnetic ions [3]. Such efficiency comes from the fact that this is a nondestructive analysis, which facilitates the coupling with other techniques [1–4]. Furthermore, operation and maintenance costs of the equipment are low, compared with current techniques for quantification.

This technique is performed with the known CPMG (Carr-Purcell-Meiboom-Gill) pulse sequence which measures the transverse relaxation time constant (*T*_2_) of the ^1^H nuclei. When in an aqueous solution, the unpaired electrons of the paramagnetic ions interact with the ^1^H nucleus in the solvent accelerating the relaxation process [5–8]. The variation of *T*_2_ is inversely proportional to the concentration of the paramagnetic ions. This allows the construction of a calibration curve. This technique is known as relaxometry.

The objective of this work was to compare the relaxometry technique, for the quantification of the paramagnetic ions contained in aqueous electrolytic solutions, using the UV-Vis spectroscopic technique and data from the atomic absorption spectroscopy, which was obtained from the literature.

One possible application of this technique could be to monitor the process of corrosion, specifically corrosions taking place under the effect of a magnetic field, as it is already known that the magnetic field will alter the speed of the reaction [1, 2, 9].

## 2. Experimental

### 2.1. Solutions

#### 2.1.1. Cu^2+^ Solution

The stock electrolyte solution was prepared with Milli-Q water, 0.1 mol L^−1^ CuSO_4_·5H_2_O (Synth), and 0.1 mol L^−1^ Na_2_SO_4_ (ISOFAR) [1]. In order to obtain the calibration curve, this solution was diluted (using a 0.1 mol L^−1^ Na_2_SO_4_ solution) down to a concentration of 10^−5^ mol L^−1^ Cu^2+^.

#### 2.1.2. Ni^2+^ Solution

The stock electrolyte solution (Watts solution) was prepared with Milli-Q water, 300 g L^−1^ NiSO_4_·6H_2_O (Synth), 90 g L^−1^ NiCl_2_·6H_2_O (Synth), and 45 g L^−1^ H_3_BO_3_ (Fluka) [11]. The pH was adjusted to 5. This solution (Ni^2+^ 1.52 mol L^−1^) was diluted (using a 45 g L^−1^ H_3_BO_3_ solution) down to a concentration of 10^−5^ mol L^−1^ Ni^2+^.

#### 2.1.3. Cr^3+^ Solution

The stock electrolyte solution was prepared with Milli-Q water, 0.02 mol L^−1^ CrCl_3_·6H_2_O (Vetec), 10 g L^−1^ NH_4_Cl (Synth), 14 g L^−1^ NaCl (Synth), 20 g L^−1^ H_3_BO_3_ (Fluka), 30 g L^−1^ glycine (J.T.Baker), and 63 g L^−1^ methanol (Vetec) [12]. This solution was diluted (using the same solution as before, but with the absence of the chrome ions) down to a concentration of 10^−5^ mol L^−1^ Cr^3+^.

#### 2.1.4. Mn^2+^ Solution

The stock electrolyte solution was prepared with Milli-Q water, 0.1 mol L^−1^ MnSO_4_·H_2_O (Synth) water, and 0.1 mol L^−1^ Na_2_SO_4_ (ISOFAR) [13]. In order to obtain the calibration curve, this solution was diluted (using 0.1 mol L^−1^ Na_2_SO_4_ solution) down to a concentration of 10^−6^ mol L^−1^ Mn^2+^.

### 2.2. Apparatus

#### 2.2.1. Relaxometry Measurements

The measurements were performed in two equipment pieces:

- A 0.23 T, TD-NMR spectrometer (Spinlock, Córdoba Argentine).
- The home-built unilateral TD-NMR sensor (UNMR) with 0.33 T (14.2 MHz for ^1^H) constructed in a “U-shaped” geometry [14, 15].

#### 2.2.2. Spectrophotometric Measurements

The Uv/Vis spectrometer used for these experiments was from PerkinElmer manufacturer, model LAMBDA 25. The measurements were performed in the wavelength range of 1000 nm to 600 nm. The maximum absorption peaks were situated at 810, 422, and 394 nm for the aqueous solutions of Cu^2+^, Cr^3+^, and Ni^2+^, respectively.

### 2.3. CPMG Parameters

*(i) Spinlock*. The CPMG parameters were *π*/2 and *π* pulses with 6.2 and 12.4 *μ*s, respectively. The echo time (*τ*) was equal to 2000 *μ*s. The number of echoes differed for each sample but ranged from 400 to 2000 echoes. The recycling delay was 1.5 ms and 8 scans were performed.

*(ii) UNMR*. The CPMG parameters were *π*/2 and *π* pulses with 3 and 6 *μ*s, respectively. The time between each refocusing pulse (2*τ*) was 120 *μ*s, 2000 echoes were used, the recycling delay was 500 ms, and 300 scans were performed.

All measurements were performed at 25 ± 0.5°C. Seven curves were made for each ion; thereby the used value of *t*_95%_ was 2,447 (Student's *t*-test). Each calibration curve had at least 7 points.

## 3. Results and Discussion

The confidence level used for the calculation of the validation parameters for the relaxometry was 95% (Table 1). The validation parameters for the relaxometry technique, using the Spinlock and the UNMR, are shown in Table 1. To compare the relaxometry with the spectrophotometry (SP) the solutions of Cu^2+^, Ni^2+^, and Cr^3+^ were used due to their intense colors. The validation parameters for the SP are also in Table 1.

To calculate the limit of detection (LOD) and limit of quantification (LOQ) the following equations were used:

(1) LOD=X+t95%σ,LOQ=X+10σ,

where *X* is the average of the signals obtained for the blank solutions (7 measurements) and *σ* is the standard deviation. The technique's sensitivity is given by the slope of the calibration curve.

The relaxometry measurements using the Spinlock showed the best results, with a lower LOD (≈10^−6^ mol L^−1^) and LOQ (≈10^−5^ mol L^−1^) in comparison to the measurements obtained using the UNMR (≈10^−4^ and ≈10^−3^ mol L^−1^, resp.). The best efficiency of the Spinlock can be explained by the homogeneous magnetic field, which eliminates the diffusion effects that are present in the UNMR measurements. Another advantage of the Spinlock measurements is that the sample is fully analyzed while in the UNMR only a slice is analyzed (at a height of 3 mm above the sensor surface).

Despite the fact the UNMR has the highest LOQ, it has some advantages: it is an open system that allows the analysis of big samples; it is small and light (less than 2.5 kg) which facilitates its transportation and it has a larger superior limit of quantification than the Spinlock.

The sensitivity of both spectrometers is similar, differing by approximately 15%. These techniques showed the highest sensitivity towards Mn^2+^, which can be associated with the fact that the Mn^2+^ has more unpaired electrons than the other ions, which enhance the relaxation effect [5, 8]. Therefore, manganese has the lowest LOQ (≈10^−6^ mol L^−1^ for Spinlock and ≈10^−4^ mol L^−1^ for UNMR).

**Figure 1:**
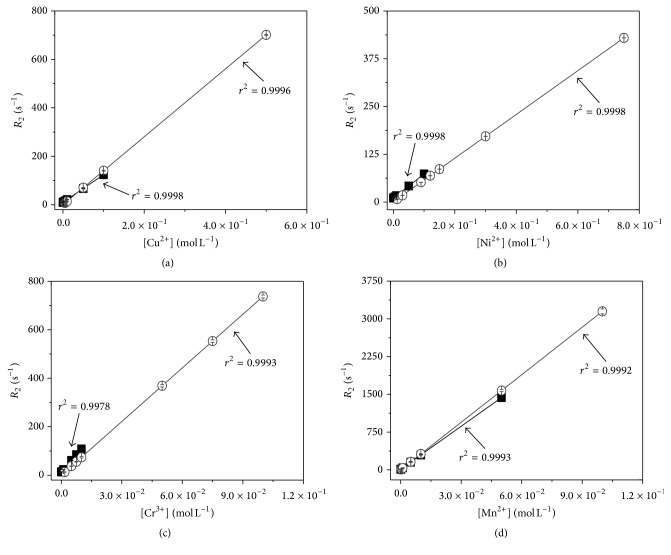
Calibration curves for relaxometry obtained using the Spinlock (it is represented by the symbol ■) and the UNMR (it is represented by the symbol ○). Each curve was made 7 times and the average is represented in the figure. The ions Cu^2+^, Ni^2+^, Cr^3+^, and Mn^2+^ are represented by (a), (b), (c), and (d), respectively.

The Spinlock and UNMR linear regression curves for ions Ni^2+^, Cu^2+^, Cr^3+^, and Mn^2+^ are shown in Figure 1. The figure is a plot of the ion concentration (mol L^−1^) versus *R*_2_ (s^−1^), which is the inverse of *T*_2_. The parameters of the regression curves are displayed in Table 2 for Spinlock, UNMR, and SP.

It is possible to note a large difference in the intercept value between both techniques (≈0.4 s^−1^ for spinlock and ≈10 s^−1^ for UNMR). This difference can be attributed to the diffusion effects that take place during the UNMR measurements. Since nothing is truly stationary in a solution and the UNMR spectrometer possesses such a large magnetic field gradient, the movement of the particles towards a different location and different magnetic field strength attenuates the measured signal [16] and the apparent relaxation time (which is different from the true relaxation time of the sample) and, thus, increases the value of the intercept of the curves.

Furthermore, by comparing the intercept value throughout the same technique but for different ions, a slight difference is noted for the Cr^3+^ ions. This difference can be explained in light of the different additives used in the chromium solution which affect the relaxation time of the sample by reducing it. This shows that the composition of the matrix must be taken into account when performing relaxometric measurements, as they can influence the relaxation time of the sample.

As expected, for (Cu^2+^)_aq_ the LOQ (≈10^−3^ mol L^−1^) for SP is higher than the relaxometry technique using Spinlock (≈10^−5^ mol L^−1^). Furthermore, the SP can be used only for quantification of compounds which have absorption bands in the UV-Visible region; because of this the (Mn^2+^)_aq_ concentration cannot be determined by this technique without a pretreatment of the sample [17].

Another technique commonly used for quantification of ions is the Atomic Absorption Spectrometry (AAS), which has the advantage of being selective, but the disadvantage is its high cost of operation and maintenance of the equipment in addition to having a limitation on the viscosity of the solvent. The LOQ for the measurements performed in the Spinlock spectrometer is approximately of one order of magnitude larger than AAS, as shown in Table 3 [10]. It is worth noting that the relaxometry technique is only sensitive to paramagnetic species and metal ions, whereas AAS is not as selective and is able to detect both metal atoms and metal ions.

Another advantage of relaxometry is that it is a nondestructive and robust technique. It can be used in solution when the solvents have high-viscosity and it does not require chemical indicators or support electrolytes [3].

A disadvantage of relaxometry is that other species in the solution, such as complexants and other paramagnetic species, can interfere with the analysis [18]. When constructing a calibration curve the matrix composition must be taken into consideration. In addition, this technique is not selective.

However, this technique has low cost of operation and maintenance, it does not require specialists for its operation, and it is a good option for use in rapid analysis and for coupling with other techniques.

A possible application of this technique is to quantify ions in residual effluents of electroplating industry and other chemical residues in a simple and fast way. The relaxometry technique could be applied to evaluate the corrosion of metallic surfaces.

## 4. Conclusions

With this work we have demonstrated that the relaxometry technique performed with the Spinlock spectrometer has a lower LOQ than the one performed with UNMR. This can be explained by the homogeneous magnetic field of the Spinlock, which eliminates diffusion effects. Nevertheless, UNMR has the advantage of being an open system, which does not limit sample size.

UNMR has proven to be better than SP, as it is able to detect paramagnetic ions without sample pretreatment, which is the case with SP when analyzing, for example, Mn^2+^ ions.

The LOQ obtained through the Spinlock spectrometer is of one order of magnitude larger than the one found with AAS (10^−5^ mol L^−1^ for Spinlock against 10^−6^ mol L^−1^ for AAS) but has the advantage of being a much cheaper and more robust technique than AAS.

In conclusion TD-NMR can be used as an alternative method to some quantification techniques, such as AAS and SP, due to its fast analyses, easy handling, and no need for sample pretreatment.

## Figures and Tables

**Table 1:** Validation parameters for relaxometry and spectrophotometry techniques.

	Ion	LOD (mol L^−1^)	LOQ (mol L^−1^)	Sensitivity^a^	Linearity range (mol L^−1^)
Spinlock	Ni^2+^	3.9 × 10^−6^	3.5 × 10^−5^	572 ± 3^b^	3.5 × 10^−5^ to 1.5 × 10^−1^
	Cu^2+^	8.5 × 10^−6^	3.5 × 10^−5^	1401 ± 8^b^	3.5 × 10^−5^ to 1.0 × 10^−1^
	Cr^3+^	3.3 × 10^−6^	1.4 × 10^−5^	7370 ± 158^b^	1.4 × 10^−5^ to 1.0 × 10^−2^
	Mn^2+^	1.1 × 10^−6^	4.6 × 10^−6^	31487 ± 282^b^	4.6 × 10^−6^ to 5.0 × 10^−2^

UNMR	Ni^2+^	3.1 × 10^−3^	1.3 × 10^−2^	632 ± 4^b^	1.3 × 10^−2^ to 7.5 × 10^−1^
	Cu^2+^	1.3 × 10^−3^	5.0 × 10^−3^	1141 ± 8^b^	5.0 × 10^−3^ to 5.0 × 10^−1^
	Cr^3+^	3.5 × 10^−4^	1.7 × 10^−3^	9470 ± 130^b^	1.7 × 10^−3^ to 1.4 × 10^−1^
	Mn^2+^	5.7 × 10^−5^	2.3 × 10^−4^	28517 ± 294^b^	2.3 × 10^−4^ to 1.0 × 10^−1^

SP	Ni^2+^	1.3 × 10^−4^	5.2 × 10^−4^	5.01 ± 0.01^c^	5.2 × 10^−4^ to 2.0 × 10^−2^
	Cu^2+^	1.0 × 10^−3^	3.6 × 10^−3^	12.46 ± 0.04^c^	3.6 × 10^−3^ to 8.0 × 10^−2^
	Cr^3+^	7.7 × 10^−4^	3.2 × 10^−3^	16.28 ± 0.29^c^	3.2 × 10^−3^ to 5.9 × 10^−2^

^a^Mean ± SD (*n* = 7); ^b^s^−1^ mol^−1^ L; ^c^cm^−1^ mol^−1^ L.

**Table 2:** Linear regression parameters for relaxometry and spectrophotometry calibration curves.

	Ion	Slope^a^	Intercept^a^	*r* ^2^
Spinlock^b^	Ni^2+^	572 ± 3	0.404 ± 0.001	0.9998
	Cu^2+^	1491 ± 8	0.402 ± 0.002	0.9998
	Cr^3+^	7370 ± 158	0.420 ± 0.010	0.9978
	Mn^2+^	31487 ± 282	0.405 ± 0.003	0.9993

UNMR^b^	Ni^2+^	632 ± 4	10.4 ± 0.1	0.9998
	Cu^2+^	1141 ± 8	10.0 ± 0.3	0.9996
	Cr^3+^	9570 ± 130	13.4 ± 0.2	0.9993
	Mn^2+^	28517 ± 294	10.3 ± 0.3	0.9992

SP^c^	Ni^2+^	5.01 ± 0.01	0.0004 ± 0.0003	0.9999
	Cu^2+^	12.45 ± 0.04	0.005 ± 0.002	0.9999
	Cr^3+^	16.28 ± 0.29	0.0365 ± 0.0513	0.9991

^a^Mean ± SD (*n* = 7); ^b^ *R*_2_ = intercept + slope[ion], where *R*_2_ (s^−1^) is the inverse of *T*_2_ and the ion concentration is given in mol L^−1^; ^c^ *A*(*λ*_810 nm_) = intercept + slope[ion], where *A* is the absorbance in 810, 422, and 394 nm for Cu^2+^, Cr^3+^, and Ni^2+^, respectively, and the ion concentration is given in mol L^−1^.

**Table 3:** Comparison between the LOQs for atomic absorption spectroscopy and relaxometry [10].

Element	AAS	Relaxometry Spinlock
	LOQ (mol L^−1^)^*∗*^	LOQ (mol L^−1^)
Ni	1.7 × 10^−6^	3.5 × 10^−5^
Cu	4.7 × 10^−7^	3.5 × 10^−5^
Cr	1.2 × 10^−6^	1.4 × 10^−5^
Mn	3.6 × 10^−7^	4.6 × 10^−6^

^*∗*^Values determined for the respective wavelengths: 232; 324.7; 537.9; and 279.5** **nm for Ni, Cu, Cr, and Mn, respectively [10].
